# Examining Delay Intervals in the Diagnosis and Treatment of Primary Open Angle Glaucoma in an Egyptian Population and Its Impact on Lifestyle

**DOI:** 10.1155/2016/7012826

**Published:** 2016-12-25

**Authors:** Iman M. Eissa, Nahla B. Abu Hussein, Ahmed E. Habib, Yasmine M. El Sayed

**Affiliations:** Ophthalmology Department, Cairo University, Giza, Egypt

## Abstract

*Purpose*. To examine causes as well as extent of delay in diagnosis and treatment of primary open angle glaucoma patients in a sample of Egyptians.* Patients and Methods*. 440 patients with primary open angle glaucoma were interviewed to evaluate delay in their diagnosis and treatment. The extent and cause of delay were investigated. The total delay interval, if any, was correlated with socioeconomic and other factors.* Results*. The median total delay was one year, with 50% of patients having a total delay of 1 year or less, of which 25% exhibited zero total delay. 25% of patients had a delay ranging from 1 to 3 years, and 25% had a total delay ranging from 3 to 27 years. Diagnostic delay accounted for 43.03% of cases. Longer delays were met in patients with certain socioeconomic factors. Patients with a positive family history of glaucoma displayed shorter delay periods.* Conclusion*. Significant delay in the diagnosis and treatment of glaucoma was found. Poor socioeconomic status seems to hinder timely diagnosis and treatment of POAG. Certain socioeconomic factors seem to correlate with the extent of delay. More effort is thus needed to subsidize the cost of investigations and treatment for glaucoma patients.

## 1. Introduction

The magnitude of glaucoma as a potentially blinding disease is continually under study. The risk of blindness from treated primary open angle glaucoma (POAG) over a period of 12–20 years is estimated to range from 14.5% to 27% in unilateral cases and from 7 to 9% in bilateral cases [1]. These figures may slightly differ in developing countries like Egypt. However, the timing of diagnosis and management of glaucoma are of crucial importance to the prognosis of the disease and its effect on the patient's lifestyle. Earlier treatment of patients will alter their mode of progression and thus may delay or totally prevent patients from reaching the stage of visual disability during their lifetime [2]. A patient with visual disability may have to resign from his job, stop driving, and/or become more dependent. Visual disability is likely once the patient reaches scale 8 on the disc damage likelihood scale (DDLS) [3].

A large percentage of glaucoma patients reside in developing countries where there are special challenges. The low socioeconomic status of most patients and the lack of facilities and scarcity of glaucoma specialists, well equipped glaucoma clinics, and screening programs may all contribute to the difficulty of timely detection of disease [4, 5].

In our glaucoma practice, we noted that a lot of patients exhibit a well established to advanced optic disc damage (DDLS 5–10) at first presentation. These patients suffered from restrictions in some of their life activities. Some patients had to change or resign from a certain job. Others stopped driving or felt dependent on other people. The authors did not find enough studies on the problem of delay in diagnosis and management of open angle glaucoma in Egypt or the Middle East [5]. This compelled the authors to conduct a study to further evaluate the causes and extent of this delay, if any.

The authors, however, found a few studies addressing the delay in diagnosis and management of other diseases like pulmonary tuberculosis in developing countries like India and Ethiopia [6, 7].

In this cross-sectional survey study, we interviewed a sample of 440 Egyptian patients, all previously diagnosed with primary open angle glaucoma (POAG) [8] with the aim of understanding the extent and causes of delay (if any) in their diagnosis and management. We also looked at the correlation between the extent of this delay and other demographic as well as socioeconomic factors. We asked the patients about restrictions in their life activities, for example, if they ever had to change or resign from a job or stop driving after being diagnosed with glaucoma or as a sequel of poor vision or poor visual field.

## 2. Patients and Methods

Five hundred and thirty Egyptian patients with a confirmed diagnosis of primary open angle glaucoma according to the International Society of Geographical and Epidemiological Ophthalmology (ISGEO) classification [8] were initially approached at the glaucoma clinic of Cairo University Hospital (Kasr Al-Ainy) from June 2012 till January 2015. Of these, 28 patients declined participating in the study. Sixty-two patients were willing to participate in the study but were excluded mainly for being unable to provide clear data as regards the period of delay and its causes (58 patients), as well as not wishing to answer the full questionnaire including data related to their socioeconomic status (4 patients).

Four hundred and forty participants eventually took part in this study. Informed consent was taken from all patients. The study adhered to the guidelines of the declaration of Helsinki [9] and was approved by the institutional ethics committee.

Cases of open angle glaucoma were diagnosed in accordance with the International Society of Geographical and Epidemiologic Ophthalmology (ISGEO) classification [8]. Accordingly, primary open angle glaucoma was classified based on three levels of evidence into three categories. The first category is based on the presence of structural and functional evidence. It requires a CDR or CDR asymmetry ≥97.5th percentile (CD 0.7) of the normal population with a visual field defect that is consistent with glaucoma. The second category included patients with advanced structural damage and unproven visual field loss. It included those subjects in whom visual field testing could not be performed or yielded unreliable results, with a CDR or CDR asymmetry ≥99.5th percentile for the normal population (CDR 0.85, CDR asymmetry 0.3). The third category consisted of patients with an IOP ≥99.5th percentile (CDR 0.85) of the normal population, whose optic discs could not be assessed due to media opacities. POAG was diagnosed if a subject fell under any of the three categories in the presence of an open and normal appearing angle on gonioscopy [8].

Inclusion criteria were POAG patients, above 20 years of age, who were on regular antiglaucoma medications or who underwent argon laser trabeculoplasty (ALT) or glaucoma surgery and who were coming for follow-up in our glaucoma clinic.

Exclusion criteria were patients with other types of glaucoma (chronic angle closure, pigmentary glaucoma, pseudoexfoliation, or any secondary open angle glaucoma). Patients who could not remember when they were first diagnosed with POAG or when they started medications, patients who wished to keep their data personal, and/or those with a documented psychiatric condition which interfered with taking the questionnaire were also excluded from the study.

The authors met two types of glaucoma patients; type A patients who had symptoms that were likely caused by glaucoma (like visual field defects consistent with glaucoma) and who consequently sought ophthalmological advice and were eventually diagnosed with POAG and type B patients who were opportunistically discovered during routine medical checkup or those who presented to an ophthalmologist with a complaint that is mostly unrelated to glaucoma (e.g., to renew their glasses, to treat conjunctivitis, or for LASIK assessment) and were advised to be investigated for glaucoma.

One-to-one in-depth interview was held with each patient during one of his/her follow-up visits. Patients were thoroughly interviewed about the history of their disease. The patient was asked if he/she can clearly state when was the very first time that he/she sought ophthalmological advice (in type A patients) or the first time that he/she was told there was a suspicion of glaucoma (in type B patients). The patient was then asked when his/her diagnosis was confirmed for glaucoma, and since when he/she was on antiglaucoma medications or underwent ALT or glaucoma surgery.

The period between the very first appearance of a problem (symptoms in type A patients or suspicion of POAG in type B patients) and the date of initiation of antiglaucoma therapy (whether medical or interventional) was calculated in years—or fraction of years—and was recorded as the* “total delay*.” Patients in whom the total delay did not exceed one month (0.08 years) were considered to have zero total delay. Since typically it takes up to a month for a newly diagnosed glaucoma patient in Egypt to have the required investigations done, book a follow-up appointment, and get started on antiglaucoma medications.

**Figure 1:**
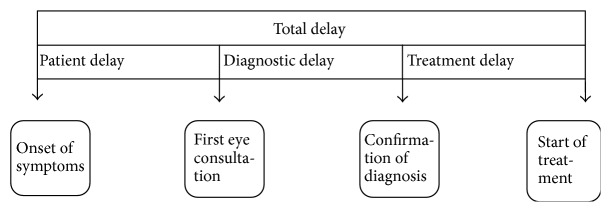
Conceptual diagram on the definition of delays, adapted from Yimer et al. [7].

The patient was then further asked about the predominant reason for this delay. The predominant cause of delay* (the type of delay)* was further classified by the authors as either patient, diagnostic, or treatment delay (Figure 1).* Patient delay* was defined as the time between the onset of a complaint (in type A patients) and the patient's first presentation to an ophthalmologist. Type B patients who were discovered opportunistically were considered to have zero patient delay and were opt to be evaluated only for “diagnostic and/or treatment delay” because these patients' delay cannot be attributed to them as they had no ocular complaint.

*Diagnostic delay* was defined as the interval between the first consultation with an ophthalmologist and the confirmed diagnosis of glaucoma.* Treatment delay* was defined as the interval between the confirmed diagnosis of glaucoma and the actual initiation of therapy whether topical medications, ALT, or surgery whichever came first.

All questions were asked by the same investigator and each interview took about twenty minutes. The interviewer asked the patients in a relaxed atmosphere, posing the questions in a nonleading, open discussion manner. The interviewer stressed on the importance of receiving accurate information rather than just getting all his questions answered and encouraged the patient to inform him of not being able to give an accurate answer rather than speculating. The patients were asked to bring or show any supportive documents (like old prescriptions, old field test printouts, or requests for investigations) with a date on them which helped to further validate the dates that they reported.

To further understand the reasons behind this type of delay, further questions were asked about the cause which has led to this type of delay. The exact cause behind a certain type of delay was enquired about. The patient was also asked if he had encountered any restrictions on his daily activities, for example, if he had to resign from or change a certain job or stop driving or cycling because of his visual disability.

After the “cause of delay” was thoroughly investigated, the patient was then asked about his education level (classified by authors into the following: illiterate, finished elementary education, middle or high school, and having a university degree or higher), the presence or absence of a family history of glaucoma, the presence of an associated systemic disease (diabetes, hypertension, ischemic heart disease, or others), whether he had enough knowledge about the risk and possible consequences of glaucoma, and finally whether the patient is covered by medical insurance or not (whether partially or totally). The participant's age, sex, and laterality of disease were also recorded for all patients. Statistical correlations were then examined between the extent of total delay and all these socioeconomic factors.

Data were statistically described in terms of mean ± standard deviation (±SD), median, range and percentiles, or frequencies (number of cases) and percentages when appropriate. Correlation between various variables was done using Spearman rank correlation equation. A *P* value less than 0.05 was considered statistically significant. All statistical calculations were done using computer program SPSS (Statistical Package for the Social Science, SPSS Inc., Chicago, IL, USA).

## 3. Results

Our study included 143 (32.5%) females and 297 (67.5%) males with a mean age of 52.05 ± 8.42 years.

The mean total delay was 2.31 ± 3.51 years, ranging from zero to 27 years. The median total delay was one year with half the number of patients falling below and the other half falling above a total delay of one year.

**Figure 2:**
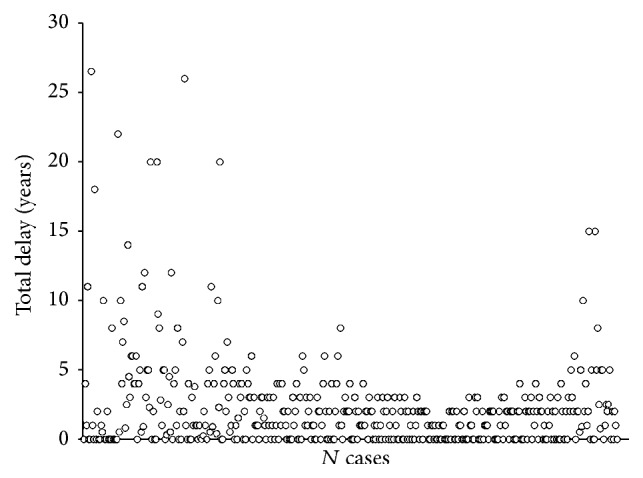
Distribution of total delay in years (*y-*axis) among number of cases (*x-*axis).

The twenty-fifth percentile was found to be at zero years (i.e., 25% of patients had zero total delay), the 50th percentile was at one year (50% of patients showed a total delay of 1 year or less), the seventy-fifth percentile was at 3 years with 75% of patients showing a delay of 3 years or less, and the last 25% of patients showed a total delay between 3 and 27 years. Interquartile range (IQR) ranged from 1 to 36 months.  Figure 2 shows the individual distribution of total delay in years among our 440 patients.

Upon further analysis of data, we found that, out of the 330 patients (75%) who exhibited a positive total delay, “patient delay” accounted for 56 patients (16.96%) “diagnostic delay” for 142 patients (43.03%), and “treatment delay” for 132 patients (40%).

### 3.1. Upon Analyzing Causes of “Patient Delay”

21.05% were type A patients who had poor vision and/or a visual field defect which they initially ignored. 78.95 % were type B patients who were advised to seek an ophthalmologist for suspicion of glaucoma. Of these, 10.52% had miscellaneous personal reasons for not promptly seeking specialized eye care, like being pregnant and preferring to wait for delivery first, working abroad or repeated business travel, and being on a waiting list to see a special doctor, and some said they had personal issues that they did not want to reveal. The remaining 68.43% stated that they simply ignored a doctor's advice to see a specialized ophthalmologist for suspected glaucoma because they did not think it was a serious problem.

### 3.2. The Main Reasons for Diagnostic Delay

The main reasons for diagnostic delay were inability to afford the cost of investigations needed to confirm the diagnosis (36.96%), delay in performing the needed investigations due to prolonged paper work with medically insured patients (36.96%), controversial doctor's opinions in patients who preferred to take a second opinion (17.39%), and patients with ocular hypertension (OHT), suspicious cupping, or visual field defects, who later progressed into glaucoma and were not timely diagnosed at the transition into actual glaucoma that required treatment (8.69%).

### 3.3. The Main Reasons for Treatment Delay

The main reasons for treatment delay were inability to afford the cost of medications and other therapy forms (68.3%) and patient delay in the execution of the prescribed treatment regimen due to ignorance and/or nonadherence or poor explanation of the importance of treatment by the treating doctor (31.7%).

Upon correlating the extent of total delay period with the patients' age, sex, and other socioeconomic factors we found the following (Table 1).

#### 3.3.1. Age and Sex of the Patient

A positive correlation was found between the extent of total delay in years and the age of the patient (*r* = 0.438), which was statistically of high significance (*P* < 0.001). However, no significant correlation was found (*r* = 0.058) between the patient's sex and the extent of delay in years (*P* = 0.225).

#### 3.3.2. Level of Education and Knowledge about the Disease

A negative correlation of high statistical significance (*P* < 0.001) was found between the level of education of patients and the total delay in years, with the delay increasing the less the patient's education level (*r* = −0.366).

Another highly significant (*P* < 0.001) negative correlation was found between the patient's knowledge about glaucoma as a disease and its possible sequelae and the delay in years. Again longer delays were met in patients with poor knowledge about glaucoma (*r* = −0.283).

#### 3.3.3. Laterality of Glaucoma and the Presence of Associated Systemic Disease

No statistically significant correlation (*r* = 0.030) was found between the disease being unilateral or bilateral and the delay in years (*P* = 0.533). However, a statistically highly significant (*P* < 0.001) positive correlation was found between the presence of associated systemic disease and the extent of delay in years (*r* = 0.219).

#### 3.3.4. Family History of Glaucoma and the Presence of Medical Insurance

A highly significant negative correlation (*P* < 0.001) was found between having a positive family history of glaucoma and the extent of delay in years, with patients exhibiting shorter delay if they had a positive family history of glaucoma (*r* = −0.305). Another statistically significant (*P* = 0.010) weak positive correlation (*r* = 0.122) was found between having a medical insurance and the delay in years with patients having medical insurance exhibiting longer delay intervals. Table 1 summarizes the correlations between the total delay in years and demographic as well as other socioeconomic variables.

Of our 440 interviewed patients, 14 patients (3.18%) had to quit their job or change it to a less visually demanding one. Twenty-nine patients have stopped driving cars, buses, or tricycles (6.59%). Most patients gave positive complaints about becoming less independent at home, a complaint which was not considered statistically because of its possible psychological origin (false sense of insecurity).

## 4. Discussion

The mean total delay in this study was 2.31 ± 3.51 years. The median total delay was one year, and interquartile range (IQR) was 1–36 months. The main type of delay in our study was diagnostic delay, where it took patients longer than usual to get an accurate and confirmed diagnosis of glaucoma. This has an impact on the prognosis of disease. Our results which showed a positive delay in 75% of our cases were similar to those reported in a study done in Iran on 258 newly diagnosed glaucoma patients [5]. However, this study focused on low socioeconomic status effect on the severity of disease at initial presentation and not on the types and causes of delay. Socioeconomic factors seem to have a direct impact on the prognosis of chronic diseases, including glaucoma [10–14].

In our study, a main cause of both diagnostic and treatment delay in POAG patients was financial incapacity, either to perform the investigations needed (36.96%) or to buy the required medication (68.3%). This also agrees with the previous studies.

Our main type of delay was diagnostic delay, being responsible for 43.03% of patients who suffered delay. We presume that the lack of medical insurance in most patients would account for this delay in diagnosis, as the cost of initial investigations is high. However, we found that 36.96% of our patients who had diagnostic delay complained of prolonged paper work which delayed the course of diagnosis despite the fact that they were medically insured. It seems that having medical insurance may contribute to delay by the prolonged paper work in our healthcare system.

Developing countries often have highly heterogeneous healthcare delivery system, with both public and private sector healthcare providers. Patients tend to move from one provider to another before they are finally properly diagnosed by a specialist and given proper treatment [6, 15, 16]. This was also true with our patients, who spent some time looking for the right eye specialist and/or whose diagnosis was sometimes delayed due to controversial doctors' opinions from different healthcare providers. The patient finally settles with the specialist whom he follows up with but some time may elapse until this is attained.

Delay in the diagnosis and treatment of POAG seemed to affect our patients' lifestyle as well, with 9.77% of our patients having had to either resign from or change a job or stop driving vehicles as a result of their visual impairment.

Upon studying the correlations between the extent of total delay in years and patient's socioeconomic factors, we found that higher patient education level, a positive family history of glaucoma, and knowledge about the nature of glaucoma were all associated with shorter periods of delay, as opposed to increasing patient's age, the presence of associated disease, and the presence of medical insurance which seemed to be associated with longer delay periods. These findings agree with what other studies found that patient awareness and level of education are important factors for prompt diagnosis and management of disease [17]. A higher male-to-female ratio was noted in our sample population. Whether that represents a true gender difference in disease distribution or is just due to males having easier access to healthcare providers due to cultural factors needs to be further investigated.

However, there are limitations to our study. POAG, unlike other diseases like tuberculosis or cancer, is largely initially asymptomatic inevitably leads to some sort of “natural delay” so long as the patient is not yet discovered. Besides, being a questionnaire based study, there is a chance of some recall bias among patients. However, we tried to minimize that by excluding any patients who did not give clear-cut dates about their delay period (at least with respect to number of years and months). We believe that the results we got can still contribute to evaluating the magnitude of the problem of delay in POAG diagnosis and treatment in our society.

Since the high costs of investigations and treatment were found to be major obstacles leading to diagnostic and treatment delay in our study, the authors recommend that more money should be spent subsidizing treatment of glaucoma patients. More efforts should be done to provide adequate medical insurance minimizing the steps involving paper work so the patients can be promptly put on track for glaucoma diagnosis and treatment. Glaucoma awareness programs should have more weight in the media and in clubs, youth organizations, and universities. Explanatory posters should be hanged and seminars held to educate the general population about glaucoma and its long term effects as this shall decrease possible future patient delay.

## Figures and Tables

**Table 1:** Correlations between delay in years and different variables.

	Delay in yrs.
Spearman's rho	
Sex	
Correlation coefficient	0.058
*P* value	0.225
*N*	440
Age	
Correlation coefficient	0.438
*P* value	0.000
*N*	440
Education level	
Correlation coefficient	−0.366
*P* value	0.000
*N*	440
Bilaterality	
Correlation coefficient	0.030
*P* value	0.533
*N*	440
Family history	
Correlation coefficient	−0.305
*P* value	0.000
*N*	440
Associated disease	
Correlation coefficient	0.219
*P* value	0.000
*N*	440
Knowledge about glaucoma	
Correlation coefficient	−0.283
*P* value	0.000
*N*	440
Insurance	
Correlation coefficient	0.122
*P* value	0.010
*N*	440
